# The Public Health Burden of Cardiomyopathies: Insights from a Nationwide Inpatient Study

**DOI:** 10.3390/jcm9040920

**Published:** 2020-03-27

**Authors:** Simon Lannou, Nicolas Mansencal, Cécile Couchoud, Mathilde Lassalle, Olivier Dubourg, Bénédicte Stengel, Christian Jacquelinet, Philippe Charron

**Affiliations:** 1 APHP, Service de Cardiologie, Centre de référence des maladies cardiaques héréditaires ou rares, Hôpital Ambroise Paré, 92100 Boulogne Billancourt, France; simonlannou@gmail.com (S.L.); nicolas.mansencal@aphp.fr (N.M.); olivier.dubourg@aphp.fr (O.D.); 2 Univ Paris-Saclay, Univ Versailles-Saint Quentin, Univ Paris-Sud, Inserm, Clinical Epidemiology Team, CESP Centre for Research in Epidemiology and Population Health, 94807 Villejuif, France; benedicte.stengel@inserm.fr (B.S.); christian.jacquelinet@biomedecine.fr (C.J.); 3 Agence de la Biomédecine, 93212 Saint-Denis la Plaine, France; cecile.couchoud@biomedecine.fr (C.C.); mathilde.lassalle@biomedecine.fr (M.L.); 4 APHP, Département de Génétique, Centre de référence des maladies cardiaques héréditaires ou rares, Hôpital Pitié-Salpêtrière, 75013 Paris, France; 5 Sorbonne Université, INSERM, UMR_S 1166 and ICAN Institute for Cardiometabolism and Nutrition, 75013 Paris, France

**Keywords:** cardiomyopathy, epidemiology, prevalence, heart transplant, defibrillator, hospitalization

## Abstract

Cardiomyopathies are responsible for heart failure and sudden cardiac death, but epidemiological data are scarce and the public health burden may be underestimated. We studied aggregating data from all public or private hospitals in France. Patients were categorized from relevant ICD-10 codes into dilated, hypertrophic, restrictive, or other cardiomyopathies (DCM, HCM, RCM, or OCM, respectively). Between 2008 and 2015, a total of 326,461 distinct patients had cardiomyopathy-related hospitalizations. The hospital-based prevalence of cardiomyopathy was 809 per million inhabitants (PMI) per year, including 428 PMI for DCM, 101 PMI for HCM, 26 PMI for RCM, and 253 PMI for OCM. Patients with cardiomyopathies accounted for 51% of all heart transplants, 33% of defibrillator implantations, 38% of mechanical circulatory supports, and 11.3% of hospitalizations for heart failure. In patients less than 40 years of age, these figures were 71%, 51%, 63%, and 23%, respectively. Over 2008–2015 and considering all cardiomyopathies, there was a significant increase for heart transplant (average annual percentage change, AAPC: +3.86%, *p* = 0.0015) and for defibrillator implantation (AAPC: +6.98%, *p* < 0.0001), and a significant decrease of in-hospital mortality (AAPC: −4.7%, *p* = 0.0002). This nationwide study shows that cardiomyopathies constitute an important cause of hospitalization, with increasing invasive therapeutic procedures and decreasing mortality.

## 1. Introduction

Cardiomyopathies are defined as myocardial diseases with abnormal structure and function in the absence of coronary disease, hypertension, valvular heart disease, or congenital malformation sufficient to explain them [1,2,3,4]. Cardiomyopathies are usually divided into four main subtypes [1]: Dilated cardiomyopathy (DCM) [5], restrictive cardiomyopathy (RCM) [6], hypertrophic cardiomyopathy (HCM) [7], arrhythmogenic right ventricular cardiomyopathy (ARVC) [8], and other nonclassified subtypes.

These diseases are considered to be significant causes of heart failure and sudden cardiac death, at least in the young [4,9,10]. However, epidemiological data are scarce, which makes it difficult to identify the burden of these diseases in public health and thus identify priorities for action at the national or international level. The rare data mainly assess the prevalence of some cardiomyopathy subtypes, or cardiomyopathies and myocarditis, in the general population [10,11,12], or among the causes of death [10,13] or sudden cardiac death in the young [14,15]. Previous studies observed a prevalence of 1/500 for HCM [11] and ~1/2500 for DCM [12] in the general population, but these could be underestimates [13]. A European prospective registry was recently conducted on the various cardiomyopathies [16], but this EURObservational Research Programme (EORP) project was restricted to selected centers and was thus unable to estimate population prevalence.

The goal of the present study was to assess the public health burden of cardiomyopathies in terms of hospitalizations, therapeutic procedures, and their trend over time through a nationwide study. Our hypothesis was that cardiomyopathies account for an underestimated part of cardiovascular morbidity, notably through hospitalizations for acute events or for invasive therapeutic procedures.

## 2. Materials and Methods

### 2.1. National Inpatient Database

The Diagnosis Related Groups (DRG) national database compiled by the Technical Agency for Information on Hospitalization (ATIH) in France aggregates data from all public and private hospitals supplying the “Program of Medicalization of the Information Systems” (PMSI) and DRG payment system [17].

Confidential and anonymous data were retrieved via the ATIH server from 2008 to 2015. For each hospitalization, a standardized discharge summary (SDS) was produced, containing a “main diagnosis,” “connected diagnosis” (on the grounds of the stay), “associated diagnoses,” and medical procedures performed during hospitalization. Diagnoses were encoded using the International Classification of Diseases version 10 (ICD-10), whereas procedures were encoded using a specific nomenclature referred to as “Classification Commune des Actes Médicaux” (CCAM).

### 2.2. Population

All patients hospitalized at least once in France during the study period were included. All hospitalization modalities were considered (day hospital, conventional hospitalization, intensive care unit). To focus on the epidemiological burden of cardiomyopathy, we restricted our study to inpatient major cardiovascular events and procedures reasonably related to a cardiomyopathy, including clinical events (such as heart failure) and invasive therapeutic procedures (such as defibrillator implantation), but we excluded other reasons, such as acute coronary syndrome.

### 2.3. Diagnostic Codes and Longitudinal Derivation Rules

We combined the relevant ICD-10 codes available for cardiomyopathies to categorize patients into the following four subgroups: DCM, HCM, RCM, and other cardiomyopathies (OCM, including ARVC and other nonclassified subtypes that do not have a dedicated disease code) (Supplementary Table S1).

To avoid bias related to encoding, to have more specific populations, and in order to minimize the risk of misclassification into cardiomyopathies, we used a cautious and conservative approach by making a restrictive choice for some ICD-10 or SDS native codes, excluding patients with another cause/code of heart disease across the study period (possibly due to comorbidity, but also due to inappropriate or wrong coding) and refined our groups of cardiomyopathies. Since about 50% of patients labelled as “DCM disease code” were also reported with an “ischemic heart disease code,” we decided to remove all patients with ischemic heart disease coding (I20 to I25 codes) from DCM in order to have a more specific DCM group. Similarly, we decided to remove all patients (about 20%) with the following coexisting disease codes: aortic stenosis, hypertensive heart disease, and arterial hypertension (I350, I352, I060, I062, I110, and I119 codes), from the HCM group. 

Each clinical event driving the hospitalization (such as heart failure) was coded as the main diagnosis or an associated diagnosis for the hospitalization (Supplementary Table S1). Every patient with the association of a “cardiomyopathy” code and a clinical event code (such as heart failure) was counted as a (heart failure) clinical event. Each medical procedure (diagnostic or therapeutic) performed during hospitalization has a CCAM code assigned to the associated hospitalization (such as cardiac transplantation). CCAM codes are detailed in Supplementary Table S2.

We also used the CRISTAL national registry, the dedicated registry for all transplantations in France, including heart transplants (HTx) [18], as an external control to validate the methodology we used for PMSI diagnostic code derivation rules. For each heart transplant performed in France between 2008 and 2015, we studied the etiology underlying HTx in CRISTAL and then compared heart transplants over the same period in the two databases (DRG/PMSI and CRISTAL).

### 2.4. Statistical Analyses

The analysis of the DRG/PMSI data was performed with the SAS Guide V7.1 computer software (SAS Institute Inc., Cary, NC, USA) by accessing the ATIH server. The annual inpatient prevalence of cardiomyopathy diagnosis, or cardiovascular event, was defined as the proportion of mainland France inhabitants with at least one hospitalization with a code of “cardiomyopathy” in a given year and expressed per million inhabitants (PMI) per year. Population estimates by age and gender were provided by the French office for national statistics (INSEE) as of 31 December for each year (Supplementary Table S3). Only one hospitalization per patient per year was considered. To assess trends over time in inpatient prevalence of cardiomyopathies, we provided the percentage change between 2008 and 2015 and the average annual percentage changes (AAPC) with their 95% confidence interval computed using the Poisson regression in the Joinpoint regression program (version 4.5) [19]. A *p*-value < 0.05 was considered statistically significant.

## 3. Results

### 3.1. Inpatient Prevalence and Trend of Cardiomyopathies

Out of 97.3 million hospitalizations in France between 2008 and 2015, a total of 326,441 distinct patients experienced at least one cardiomyopathy-related hospitalization (Supplementary Figure S1). These patients had a mean age of 71 years and were predominantly males (62%). Over 2008–2015, the annual inpatient prevalence (DRG-mean annual prevalence) of overall cardiomyopathy was 809.5 ± 29.1 (mean ± sd) per million inhabitants (PMI). With regard to cardiomyopathy subtypes (Table 1), the prevalence was mainly driven by patients with DCM (428.4 PMI, 53% of all patients), followed by the OCM group (253.4. PMI, 31%), HCM group (100.8 PMI, 12%), and RCM group (25.9 PMI, 3%). The total inpatient prevalence of cardiomyopathies did not change significantly between 2008 and 2015 (844 PMI in 2008 and 847 PMI in 2015, average annual percentage changes or AAPC: +0.2%, [95% CI: –0.16; 0.57], *p* > 0.05), with an increase of DCM and RCM but a decrease of HCM and OCM. The increase was particularly high for RCM (+48% between 2008 and 2015, 21.9 PMI to 32.4 PMI). The numbers standardized for age and sex are provided in Supplementary Table S3.

### 3.2. Cardiomyopathy-Related Cardiovascular Events and Invasive Procedures

From 2008 to 2015, a total of 233,872 distinct patients were hospitalized at least once for a cardiovascular event or a therapeutic procedure directly related to the cardiomyopathy (Supplementary Figure S1). The most common nonfatal event was heart failure, followed by stroke, arrhythmia, pulmonary embolism, and endocarditis. The most common invasive procedure was the implantation of a pacemaker, then a cardiac defibrillator and heart transplant. A total of 44,585 patients died in hospital with a related diagnosis of a cardiomyopathy.

**Figure 1:**
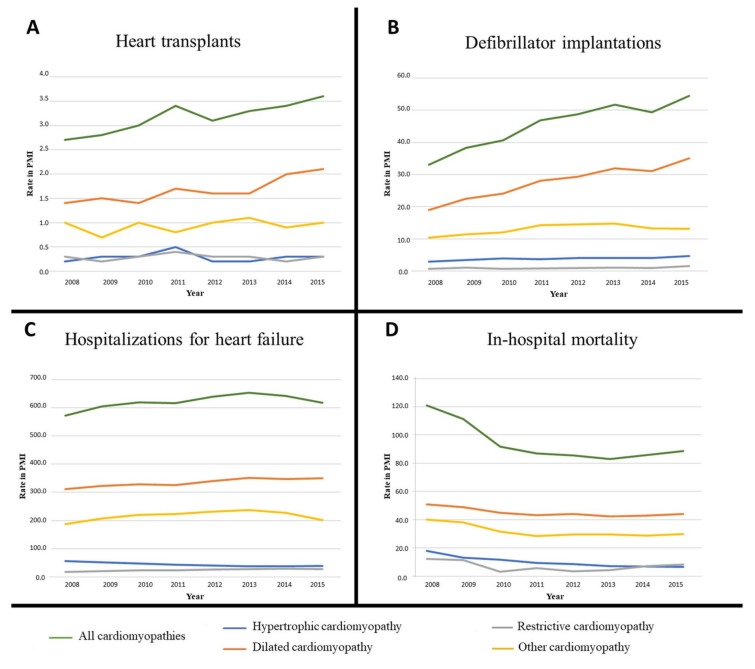
Temporal variations of inpatient prevalence of cardiomyopathy patients with at least one clinical event or procedure from 2008 to 2015: **A.** Heart transplants; **B.** Defibrillator implantations; **C.** Heart failure; **D.** In-hospital mortality. PMI: Per million inhabitants.

Temporal variations of the prevalence of patients who underwent at least one clinical event or an invasive procedure over the study period are detailed in Table 2 and Table 3, as well as in Figure 1, by event or procedure and by cardiomyopathy subtype. Over 2008–2015 and considering all cardiomyopathy subtypes together, there was a nonsignificant increase in the prevalence of inpatients heart failure (average annual percentage change or AAPC: +0.94%, (95% CI: –0.7;2.6), *p* > 0.05), a significant increase for heart transplant (AAPC: +3.86%, (2.13;5.61), *p* = 0.0015), for defibrillator implantation (AAPC: +6.98%, (3.68;10.38), *p* < 0.0001) and a significant decrease of in-hospital mortality (AAPC: –4.7%, (–7.09;–2.26), *p* = 0.0002). Additional results, including the comparison of heart transplants in the CRISTAL database, are mentioned in Supplementary Text S1.

### 3.3. Proportion of Cardiomyopathies among all Cardiovascular Hospitalizations

**Figure 2:**
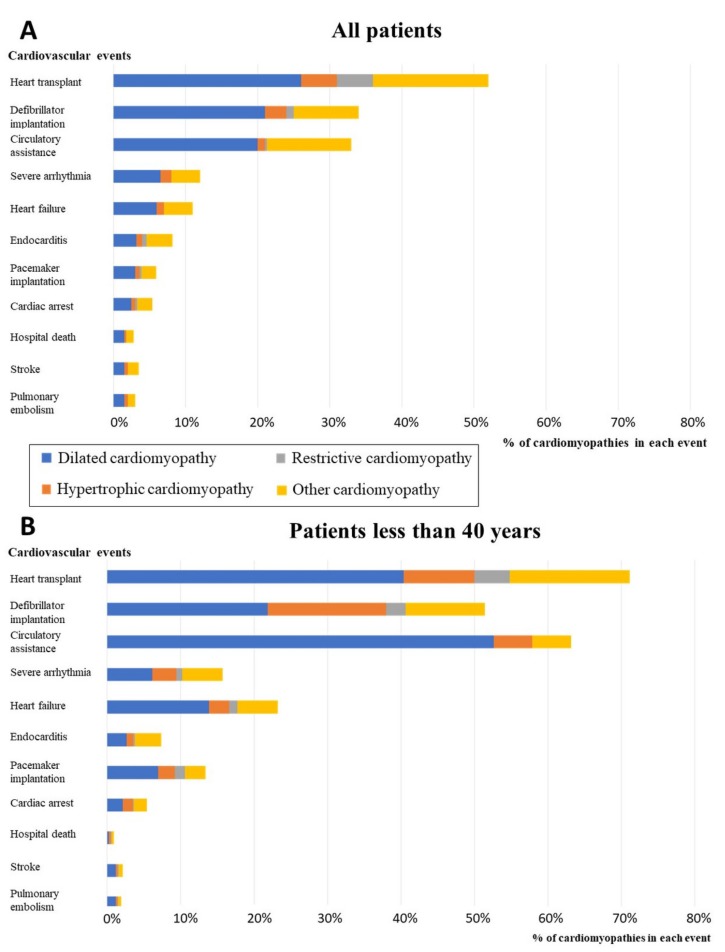
Proportion of patients with cardiomyopathies among all patients hospitalized for at least one major cardiovascular event or invasive therapeutic procedure in 2015. **A**. Proportions in the total population. **B**. Proportion in the subgroup of patients less than 40 years of age. Cardiac arrest: includes codes for sudden death, cardiac arrest, and aborted cardiac arrest.

We observed that cardiomyopathies accounted for a significant proportion of hospitalizations related either to invasive cardiac therapeutics or to major cardiovascular events (MACE) (Figure 2). When considering all procedures or MACE in France in 2015, whatever the underlying cardiac disease and including coronary artery diseases, we observed that cardiomyopathies (all subtypes considered together) accounted for 51% of all heart transplants, 33% of defibrillator implantations, 38% of mechanical circulatory assistance, 7% of pacemaker implantations, 11.3% of heart failures, and 13% of severe ventricular arrhythmias. We found cardiomyopathies as comorbidities in 8% of infectious endocarditis cases, 5% of cardiac arrests, 4% of strokes, and 3% of pulmonary embolisms and in-hospital deaths. For all these cardiovascular events, DCM was the most prevalent contributing cardiomyopathy subtype (Figure 2A).

Moreover, when considering only the patients less than 40 years of age (Figure 2B), cardiomyopathies account for 71% of all heart transplants, 51% of ICD implantations, 63% of circulatory assistance, 13% of pacemaker implantations, 23% of heart failures, 16% of severe arrhythmias, 7% of infectious endocarditis cases, 5% of cardiac arrests, 2% of strokes, 2% of pulmonary embolisms, and 1% of in-hospital deaths. For all these cardiovascular events, DCM was the most prevalent contributing cardiomyopathy subtype.

## 4. Discussion

This study is one of the few studies conducted on the epidemiology of cardiomyopathies and the first detailed study of a nationwide health burden of cardiomyopathies in terms of cardiovascular hospitalizations and invasive therapeutic procedures.

The first result from this exhaustive national study is that, overall, with all subtypes of cardiomyopathies combined, cardiomyopathies constituted an important part of cardiovascular hospitalizations and invasive therapeutic procedures. Cardiomyopathies constituted 51% of all cardiac transplants, ahead of ischemic heart disease, which is consistent with several national heart transplantation registries [20,21,22], indicating that cardiomyopathies, especially dilated cardiomyopathy, have now exceeded coronary artery disease as the major indication, possibly in relation to the better management and prognosis of myocardial infarction. Interestingly, we were also able to show that cardiomyopathies constituted a third of defibrillator implantations and a third of circulatory assistances, which was not previously reported and further emphasizes the global health burden of these diseases. Cardiomyopathies also accounted for a significant proportion of hospitalizations for heart failure (11%), similar to a figure reported in the U.S. [23], and a significant proportion of severe ventricular arrhythmias (13%). Moreover, the proportions were even larger when considering only the patients under 40 years of age (cardiomyopathies accounted for 71% of all heart transplants, 51% of all ICD implantations, 63% of circulatory support, and 23% of heart failures). Overall, all subtypes of cardiomyopathies combined and all patients considered, whatever their age, the prevalence of hospitalizations for distinct patients with a cardiomyopathy was estimated to be 847 per million inhabitants in 2015 (55,000 independent patients each year in France), which is more than the prevalence observed for several severe diseases such as pulmonary hypertension [24]. Moreover, over the study period (from 2008 to 2015), we observed a significant trend toward further recent increase in the prevalence per million inhabitants of hospitalizations for cardiomyopathy in relation to heart transplant (+33%) or defibrillator implantation (+66%). The explanations might be related to more proactive therapeutic indications, due to extended recommendations [25]. At the same time, we observed a reduction of in-hospital mortality (–27.3%) that might be related to the increase of therapeutic procedures.

When considering the respective weights of cardiomyopathy subtypes, patients with DCM were predominant in terms of hospital-based prevalence over the study period (mean annual prevalence rate 428 PMI/year, 53% of all cardiomyopathy hospitalizations) with a prevalence that increased significantly from 2008 to 2015 (+13%), although median age was stable or decreased (71 years in 2008 versus 69 years in 2015). Patients with DCM were particularly predominant in hospitalizations for heart transplants (52%), defibrillator implantations (60%), and heart failures (53%). Patients with HCM represented only 12% of all cardiomyopathy hospitalizations, although the estimated prevalence of the disease in the general population is about 1/500, as compared to 1/2500 for DCM, suggesting that major cardiovascular complications are less frequent in HCM patients and require less frequent invasive therapeutics, or that the true prevalence of DCM is underestimated as suggested by some authors [26]. Furthermore, the mean age of 71 years for patients hospitalized for cardiomyopathies highlights that most of youngest patients with cardiomyopathies do not require hospitalization. Finally, we observed that RCM accounted for only 3% of all cardiomyopathy hospitalizations, in agreement with the very low prevalence of the disease in the general population [6]. However, the inpatient prevalence of RCM significantly increased over the study period (+48%), which may reflect the recent evolving knowledge about this cardiomyopathy, especially regarding transthyretin amyloid cardiomyopathy [27], and the increasing detection of these diseases through improved awareness in the medical community.

Altogether, our results show that cardiomyopathies constitute an important part of hospitalizations for invasive therapeutic procedures and major cardiovascular events. The total burden is even greater in the restricted population of patients less than 40 years of age. This proportion was not well recognized until now. Therefore, our findings may have important consequences for public health action programs. There is a definite interest in launching specific action plans for cardiomyopathies in order to improve early diagnosis, therapeutics, and prevention of event recurrence [4]. The better identification of these patients is especially important in the context of recent progress in prognosis stratification [28,29,30], along with the development of recent innovative therapeutics based on new small molecules or gene-editing approaches [31]. Our results suggest that we should focus especially on patients with dilated cardiomyopathy, who are the leading reason for hospitalizations, heart transplants, and defibrillator implantations. Our results also suggest that we should focus on patients under 40 years of age, especially to improve early diagnosis through dedicated family screening, since most (at least 40%) cardiomyopathies are familial and of genetic origin [16]. Another way to improve healthcare is to promote dedicated multidisciplinary centers of expertise on cardiomyopathies, at the national or international level, because various specific skills are required for appropriate management of these diseases.

Finally, our results may help improve diagnostic coding systems for hospitalizations at an international level. First, some patients have multiple disease diagnostic ICD-10 codes, such as dilated cardiomyopathy and ischemic heart disease, or hypertrophic cardiomyopathy and hypertensive heart disease, which may be related to comorbidities, but may also be due to misclassification or inappropriate understanding of disease definitions [32]. We decided to use a cautious and conservative approach by making a restrictive choice, excluding patients with another cause/code of heart disease. Future work may benefit from a clarification of disease definitions in the next ICD-11 or by making a clear distinction between the leading diagnostic code and a comorbidity. Second, precise diagnostic ICD-10 codes are missing for some cardiomyopathy subtypes, such as arrhythmogenic right ventricular cardiomyopathy, Takotsubo cardiomyopathy, or left ventricular noncompaction. The current diagnostic code entitled “other cardiomyopathy” may include these cardiomyopathies, but may also include other unclear phenotypes. Therefore, to promote refined diagnostic codes for cardiomyopathies, either with new codes (such as those proposed by ORPHA) [33] or by clarifying the definition of diseases (avoiding misclassification), would allow an even more comprehensive understanding of the health burden related to cardiomyopathies.

A major strength of the study was the use of a nationwide inpatient database, including detailed diagnoses and procedures from all hospitalizations, whether in the public or private sector, which enabled us to assess prevalence and trends for any type of cardiomyopathies. We also used a conservative approach about disease diagnostic codes by excluding cardiomyopathy patients with another cause/comorbidity/code of heart disease (for example, all patients with dilated cardiomyopathy *and* ischemic heart disease were classified by us as ischemic heart disease and excluded) in order to minimize the risk of misclassification and increase the specificity of the cardiomyopathy diagnosis. Therefore, we may have underestimated the true figures of patients with a cardiomyopathy. Our approach, consistent with public health objectives [34], must be read as conditionally to the derivation rules we used to control coding bias, and is a first attempt to build standardized inpatient prevalence of cardiomyopathies using the French DRG data. Interestingly, we were able to compare our results regarding heart transplantation with those from the national CRISTAL transplantation database [18] and found consistent results, although there were some minor differences that might be related to coding differences or to our derivation rules. The validity of our derivation rules remains to be evaluated but, interestingly, a meta-analysis of the validity of heart failure codes in administrative databases observed that the overall performance was good with excellent specificity but suboptimal sensitivity [35].

The study was focused on inpatient prevalence of cardiomyopathies, so we did not estimate the overall prevalence in the general population nor the total mortality rate (which should include out-hospital mortality) of patients with a cardiomyopathy. The temporal variation in number of deaths should therefore be read with caution but may indeed be an expression of the better management and outcomes of cardiomyopathies. The total prevalence of patients who have cardiomyopathies (including those who are not hospitalized) is expected to be more important, and analyzing only inpatient data may skew the prevalence of cardiomyopathies towards subtypes that account for more hospitalizations such as DCM.

Finally, the mean age of patients was relatively high, but meaningful, since only inpatients were considered (therefore with more advanced stage of the disease) and not outpatients. It might also be explained by more prolonged life durations in cardiomyopathy patients than usually thought and/or late occurrence of the disease in some cardiomyopathy subtypes, as recently suggested [16].

## 5. Conclusions

This work provides innovative data on the epidemiology of cardiomyopathies at a national level through the use of exhaustive data from medico-economic databases. We showed that cardiomyopathies constituted an important part of hospitalizations and invasive therapeutic procedures. The percentages were even greater in patients less than 40 years of age. These results may help to establish public health action programs in order to promote optimal management of these patients, especially through a better screening of these relatively frequent hereditary diseases, and further development of innovative therapeutics. Our results also highlight the need for improvement of diagnostic coding systems in hospitalizations internationally.

## Figures and Tables

**Table 1:** Inpatient prevalence of patients (PMI) with cardiomyopathies in France over the period 2008–2015.

CM	2008	2009	2010	2011	2012	2013	2014	2015	M ± sd	PC%	AAPC	IC95%	*p*-Value	%Men	Age (m)	%CM
DCM	419.9	414.5	409.1	408.5	421	435.1	446.1	472.8	428.4 ± 23.6	+13%	1.58	[0.88;2.3]	0,0000	65%	70	53%
OCM	276.6	261.3	242.6	240.3	255.4	260.3	251.1	239.6	253.4 ± 9.3	–13%	–1.1	[–2.77;0.59]	NS	57%	69	31%
HCM	124.7	109.8	101.5	95.4	89.3	89.8	93.8	101.7	100.8 ± 7.4	–18%	–2.8	[–4.39;–1.19]	0.0007	60%	70	12%
RCM	21.9	23.3	22.7	22.2	25.8	28.4	30.9	32.4	26 ± 4.1	+48%	5.9	[3.84;8]	0,0000	56%	74	3%
All CM	844.0	809.9	777.1	767.5	792.7	814.4	822.9	847.5	809.5 ± 27.6	0%	0.2	[–0.16;0.57]	NS	62%	71	100%

CM: Cardiomyopathy; DCM: Dilated cardiomyopathy; OCM: Other cardiomyopathy; HCM: Hypertrophic cardiomyopathy; RCM: Restrictive cardiomyopathy; All CM: All cardiomyopathies; m ± sd: Mean and standard deviation of DRG-AP per million inhabitants (PMI) over the 2008–2015 period; PC%: Percent change between 2008 and 2015; AAPC: Average annual percentage change on the overall period with its 95% Confidence Interval, *p*: *p*-value of having AAPC different from 0; NS: nonsignificant; %Men: Mean of annual percentages of male with specific CM; Age(m): Mean Age; %CM: Mean of annual ratios of patients with specific CM among all CM on the 2008–2015 period.

**Table 2:** Temporal variations of inpatient prevalence of events (per million inhabitants), by type of event and cardiomyopathy, over the period 2008–2015.

EVENT	CM	2008	2009	2010	2011	2012	2013	2014	2015	m ± sd	PC%	AAPC%	CI95%	*p*-Value
Heart Failure	HCM	56.2	52.5	47.1	42.7	40.1	37.5	38.1	39.4	44.2 ± 7	–30%	–4.94	[–6.17;–3.7]	<0.0001
	DCM	310.9	323.4	328.4	326	339.5	351.3	347.3	349.6	334.6 ± 14.6	12%	1.71	[1.11;2.31]	0.0004
	RCM	17.2	21.1	23	23.3	26.2	27.8	29.5	27.1	24.4 ± 4	58%	6.82	[3.84;9.89]	0.0013
	OCM	187.2	207.9	220.5	223.5	231.8	237	227	201.7	217.1 ± 16.8	8%	0.65	[–2.15;3.54]	NS
	All CM	571.9	605.5	619.5	616.4	638.5	654.1	642.5	618.3	620.8 ± 25.5	8%	0.94	[–0.7;2.6]	NS
Stroke	HCM	4.8	4.6	4.6	4.2	4.1	4.5	4.9	4.3	4.5 ± 0.3	–10%	–0.65	[–3.09;1.86]	NS
	DCM	17.8	20.2	21.7	21.9	25	24.1	24.5	24.5	22.5 ± 2.5	38%	4.25	[1.53;7.05]	0.0021
	RCM	1.4	1.6	1.8	1.9	2.1	1.9	2.5	2.5	2 ± 0.4	79%	8.11	[5.31;10.99]	0.0003
	OCM	21.7	23.4	21	20.7	21.9	22.3	22.4	19.4	21.6 ± 1.2	–11%	–0.91	[–3.02;1.25]	NS
	All	45.7	49.9	49.1	48.7	53.1	52.8	54.4	50.7	50.6 ± 2.8	11%	1.76	[0.24;3.3]	0.0299
Pulmonary Embolim	HCM	3.3	2.9	2.8	2.8	2.6	2.3	2.6	2.5	2.7 ± 0.3	–24%	–3.68	[–5.92;–1.4]	0.0078
	DCM	12.7	13.4	13.6	12.9	13.3	13.3	13.5	13.4	13.3 ± 0.3	6%	0.45	[–0.39;1.3]	NS
	RCM	0.8	1.1	1.2	1.2	1	1.2	1.3	1	1.1 ± 0.2	25%	2.67	[–3.09;8.77]	NS
	OCM	11.1	11.6	11.7	10.9	9.9	10.5	10.1	9.8	10.7 ± 0.7	–12%	–2.34	[–3.84;–0.81]	0.0098
	All	27.9	29	29.2	27.8	26.8	27.3	27.5	26.8	27.8 ± 0.9	–4%	–0.93	[–1.85;0]	0.0496
Endocarditis	HCM	0.5	0.6	0.7	0.6	0.7	0.7	0.8	0.7	0.7 ± 0.1	40%	4.81	[1.15;8.6]	0.0178
	DCM	3	3.2	3.8	3.8	3.6	4.7	4.2	3.6	3.7 ± 0.5	20%	3.91	[–0.51;8.52]	NS
	RCM	0.2	0.3	0.3	0.4	0.4	0.4	0.3	0.4	0.3 ± 0.1	100%	7.04	[–0.61;15.28]	NS
	OCM	2.4	2.8	3.6	4	4.8	4.5	4.3	4.2	3.8 ± 0.8	75%	8.01	[5.13;10.97]	<0.0001
	All	6.3	7	8.3	8.8	9.6	10.4	9.7	8.9	8.6 ± 1.4	41%	4.71	[1.68;7.83]	0.0021
Cardiac Arrest	HCM	3.1	3	3.3	2.7	2.7	2.7	2.5	2.5	2.8 ± 0.3	–19%	-3.53	[–5.48;–1.54]	0.005
	DCM	9.1	12.1	11.8	10.2	12	12.9	12.9	13.5	11.8 ± 1.5	48%	4.27	[0.74;7.92]	0.025
	RCM	0.8	1	1.2	0.9	1.2	1.4	1.7	1.4	1.2 ± 0.3	75%	9.11	[3.34;15.2]	0.0078
	OCM	7.4	8.8	9.3	10	11	11	11.1	11.6	10 ± 1.4	57%	5.82	[2.99;8.73]	0,0000
	All	20.3	25	25.7	23.8	26.9	27.9	28.1	28.9	25.8 ± 2.8	42%	4.16	[1.76;6.63]	0.0053
In-HospitalDeath	HCM	18	13.1	11.7	9.4	8.5	7.1	7	6.5	10.2 ± 3.9	–64%	–13.42	[–17.63;–9]	<0.0001
	DCM	50.8	48.8	45	43.2	44	42.3	43	44	45.1 ± 3	–13%	–2.26	[–3.87;–0.63]	0.0069
	RCM	12.3	11.4	3.3	5.7	3.5	4.2	7.1	8.4	7 ± 3.5	–32%	–10.05	[–32.58;20.01]	NS
	OCM	40	38	31.7	28.4	29.5	29.5	28.7	29.8	32 ± 4.5	–26%	–4.52	[–7.06;–1.92]	0.0007
	All	121	111.3	91.7	86.8	85.6	83.1	85.8	88.7	94.3 ± 14	–27%	–4.7	[–7.09;–2.26]	0.0002

CM: Cardiomyopathy; DCM: Dilated cardiomyopathy; OCM: Other cardiomyopathy; HCM: Hypertrophic cardiomyopathy; RCM: Restrictive cardiomyopathy; All CM: All cardiomyopathies; m ± sd: Mean and standard deviation of DRG-AP per million inhabitants (PMI) over the 2008–2015 period; PC%: Percent change between 2008 and 2015; AAPC: Average annual percentage change on the overall period with its 95% Confidence Interval, *p*: *p*-value of having AAPC different from 0; NS: nonsignificant.

**Table 3:** Temporal variations of inpatient prevalence of invasive procedure (per million inhabitants), by type of procedure and cardiomyopathy, over the period 2008–2015.

Procedure	CM	2008	2009	2010	2011	2012	2013	2014	2015	m ± sd	PC%	AAPC	CI95%	*p*-Value
Pacemaker	HCM	6.3	6.1	5.3	5.7	5	4.9	5.4	5.5	5.5 ± 0.5	–13%	–1.6	[–6.3;3.33]	NS
	DCM	31.3	31.8	30.7	27.8	27.6	26.8	25.8	25.9	28.5 ± 2.4	–17%	–3.26	[–4.25;–2.26]	0.0002
	RCM	1.9	2.2	2.2	2.1	2.5	2.4	2.6	2.6	2.3 ± 0.3	37%	4.21	[2.2;6.27]	0.0021
	OCM	18.1	18.1	19.1	18.2	18	18.2	17.8	14.7	17.8 ± 1.3	–19%	–2,00	[–4.4;0.47]	NS
	All	57.6	58.2	57.3	53.8	53.1	52.3	51.6	48.8	54.1 ± 3.3	–15%	–2.41	[–3.07;–1.75]	0.0001
Implantable Cardioverter Defibrillator	HCM	2.9	3.4	3.9	3.7	4	4	4.1	4.7	3.8 ± 0.5	62%	5.47	[2.97;8.02]	0.0016
	DCM	19	22.5	24.1	28.1	29.3	31.9	31	35.1	27.6 ± 5.4	85%	8.6	[5.01;12.31]	<0.0001
	RCM	0.7	1	0.7	0.8	0.9	1.1	0.9	1.6	1 ± 0.3	129%	8.35	[0.4;16.92]	0.042
	OCM	10.4	11.4	12	14.2	14.5	14.7	13.2	13.1	12.9 ± 1.6	26%	3.27	[0.25;6.38]	0.0334
	All	33.1	38.3	40.7	46.9	48.7	51.7	49.3	54.5	45.4 ± 7.3	65%	6.98	[3.68;10.38]	<0.0001
Heart Transplant	HCM	0.2	0.3	0.3	0.5	0.2	0.2	0.3	0.3	0.3 ± 0.1	50%	0.84	[–11.27;14.61]	NS
	DCM	1.4	1.5	1.4	1.7	1.6	1.6	2	2.1	1.7 ± 0.3	50%	5.65	[2.69;8.7]	0.0032
	RCM	0.3	0.2	0.3	0.4	0.3	0.3	0.2	0.3	0.3 ± 0.1	0%	–0.34	[–9.34;9.55]	NS
	OCM	1	0.7	1	0.8	1	1.1	0.9	1	0.9 ± 0.1	0%	2.12	[–3.5;8.08]	NS
	All	2.7	2.8	3	3.4	3.1	3.3	3.4	3.6	3.2 ± 0.3	33%	3.86	[2.13;5.61]	0.0015
Circulatory Assistance	HCM	0	0	0	0	0	0.1	0	0.1	0 ± 0	–	–	–	–
	DCM	0.2	0.2	0.3	0.4	0.4	0.5	0.9	0.6	0.4 ± 0.2	200%	22.06	[13.18;31.63]	0.0007
	RCM	0	0	0	0	0	0	0	0	–	–	–	–	–
	OCM	0.1	0.1	0.1	0.2	0.3	0.3	0.5	0.4	0.3 ± 0.2	300%	–	–	–
	All	1.2	0.9	1.2	1.5	2	2.3	2.6	3.2	1.9 ± 0.8	167%	18.71	[12.42;25.36]	0.0002

CM: Cardiomyopathy; DCM: Dilated cardiomyopathy; OCM: Other cardiomyopathy; HCM: Hypertrophic cardiomyopathy; RCM: Restrictive cardiomyopathy; All CM: All cardiomyopathies; m ± sd: Mean and standard deviation of DRG-AP per million inhabitants (PMI) over the 2008–2015 period; PC%: Percent change between 2008 and 2015; AAPC: Average annual percentage change on the overall period with its 95% Confidence Interval, *p*: *p*-value of having AAPC different from 0; NS: nonsignificant.

## Supplementary Materials

The following are available online at https://www.mdpi.com/2077-0383/9/4/920/s1, Text S1. Additional results; Table S1. ICD-10 code classification used for cardiomyopathies and for clinical events; Table S2. Codes used in France for procedures during hospitalization: CCAM or “Classification Commune des Actes Médicaux”; Table S3. Inpatient prevalence of patients with cardiomyopathies per sex and per age classes; Figure S1. Flow chart of distinct patients who experienced at least one cardiomyopathy-related hospitalization from 2008 to 2015 in France.
