# Supplementary material for: The Public Health Burden of Cardiomyopathies: Insights from a Nationwide Inpatient Study
Source: J Clin Med. 2020 Mar 27;9(4):920. doi: 10.3390/jcm9040920 (PMC7230913; doi:10.3390/jcm9040920)

Online-Only Supplements

(Supplementary text & tables)

Text S1. Additional results

Table S1. ICD-10 code classification used for cardiomyopathies and for clinical events.

Table S2. Codes used in France for procedures during hospitalization: CCAM or "Classification Commune des Actes Médicaux"

Table S3. Inpatient prevalence of patients with cardiomyopathies per sex and per age classes

Figure S1. Flowchart of distinct patients who experienced at least one cardiomyopathy-related hospitalization from 2008 to 2015 in France.

Text S1. Additional results

Heart failure

Of all cases of patients hospitalized for heart failure (HF) over the study period, cardiomyopathies accounted for 11.3%. The median age of cardiomyopathy patients hospitalized for HF was 76 years, and of patients 59% were males in 2015. The distribution of cardiomyopathy subtypes (Table 3) was as follows: 53% for dilated cardiomyopathy (DCM), 7% for hypertrophic cardiomyopathy (HCM), 1% for restrictive cardiomyopathy (RCM), and 35% for other cardiomyopathy (OCM) The temporal evolution of HF hospitalizations is described in Figure 2. The annual prevalence of patients with cardiomyopathies who experienced at least one heart failure episode increased nonsignificantly from 571 per million inhabitants (PMI) in 2008 to 618 PMI in 2015 (+8%, AAPC: +0.94(0.7;2.6), p>0.05), with a significant increase for DCM and RCM but a decrease for HCM (Table 3).

Cardiac arrest

Cardiac arrest (CA) associated with cardiomyopathies includes sudden death, cardiac arrest, and aborted cardiac arrest. Of all cases of CA, cardiomyopathies accounted for 5%. The median age of patients was 65 years, and males were predominant (62% in 2015). The distribution of cardiomyopathy subtypes was 46%

for DCM, 11% for HCM, 4% for RCM, and 39% for OCM. The prevalence of cardiac arrest in cardiomyopathy patients increased over the study period: 20.3 PMI in 2008 to 28.9 PMI in 2015 (+42%, AAPC: +4.16%, [1.76;6.63], p=0.0053). This was mainly explained by the increased proportion of cardiac arrests with successful resuscitation (9.6 PMI in 2008 to 16.7 PMI in 2015) and was observed for all cardiomyopathies.

Heart transplant

Of all heart transplants (HTx) reported in the PMSI/DRG database over the study period, cardiomyopathies accounted for the largest proportion of patients (51% of all HTx). The median age of transplanted cardiomyopathy patients was 47 years; males were predominant (70% in 2008 and in 2015). The distribution of cardiomyopathy subtypes (Table 4) was 52% for DCM, 9% for HCM, 9% for RCM and 30% for OCM. The temporal evolution of HTx is described in Figure 2. We observed that the number of HTx in patients with cardiomyopathies increased from 2.7 PMI in 2008 to 3.6 in 2015 (+33%, AAPC: +3.86%, [2.13;5.61], p<0.0015). This increase was especially observed for DCM (1.4 PMI in 2008 to 2.1 in 2015, +50%, AAPC=+5.65, [2.69;8.7], p=0.0032), whereas stability was observed for HCM, RCM, and OCM subtypes (Table 4).

According to the CRISTAL database (a national database dedicated to heart transplants), cardiomyopathies accounted for 54% of all heart transplants over the study period, whereas ischemic heart diseases accounted for 34%. The contribution of cardiomyopathy subtypes to the total number of HTx in CRISTAL was 39% for DCM (versus 27% in DRG/PMSI database), 6.9% for other (versus 15% in DRG/PMSI database), 4.4% for HCM (versus 4.5% in DRG/PMSI database), and 3.3% for RCM (versus 4.4% in DRG/PMSI database).

Automatic implantable cardioverter-defibrillator (AICD)

Of all AICDs implanted in France over the study period, cardiomyopathies accounted for 33%. The median age of cardiomyopathy patients with AICD implantation was 63 years, and males were predominant (72% in 2015). The distribution of cardiomyopathy subtypes (Table 4) was 60% for DCM, 10% for HCM, 3% for RCM, and 27% for OCM. Figure 2 describes the temporal evolution of AICD. The annual prevalence of cardiomyopathy patients who underwent a defibrillator implantation drastically increased from 33 PMI in

2008 to 55 PMI in 2015 (+65%, AAPC: +6.98%, (3.68;10.38), $p<10^{-4}$). The increase was observed for all cardiomyopathy subtypes (Table 4).

Table S1. ICD-10 code classification used for cardiomyopathies and for clinical events.

Group of cardiomyopathies	ICD 10 codes	ICD code heading
Dilated cardiomyopathy (DCM)	I420<br>I426<br>I427<br>I432	Dilated cardiomyopathies<br>OH (alcoholic) cardiomyopathies<br>Nutritional cardiomyopathies<br>Cardiomyopathies due to drugs or external causes
Restrictive cardiomyopathy (RCM)	I423<br>I424<br>I425<br>I431	Endomyocardial disease<br>Endocardial fibroelastosis<br>Restrictive cardiomyopathies<br>Cardiomyopathies in metabolic diseases
Hypertrophic cardiomyopathy (HCM)	I421<br>I422	HCM obstructive<br>HCM non obstructive
Other cardiomyopathies (OCM)	I428<br>I429<br>I430<br>I438<br>O903	Other cardiomyopathies (including Takotsubo, Arrhythmogenic right ventricular cardiomyopathy)<br>Unspecified Cardiomyopathies<br>Cardiomyopathy in infectious and parasitic diseases classified elsewhere<br>Cardiomyopathy in diseases classified elsewhere<br>Peripartum cardiomyopathy

Clinical events	ICD 10 codes	ICD code heading
Heart failure	I500<br>I501<br>I509<br>R570	Congestive heart failure (HF)<br>Left ventricular failure or acute pulmonary edema<br>Heart failure without precisions<br>Cardiogenic shock
Severe arrhythmias	I472<br>I490	Ventricular tachycardia<br>Ventricular fibrillation
Sudden death	I461	Sudden death
Stroke	I64	Stroke
Endocarditis	I33	Endocarditis
Pulmonary embolism	I26	Pulmonary embolism

Table S2. Codes used in France for procedures during hospitalization: Classification Commune des Actes Médicaux (CCAM).

Procedures	Codes
Implantation of a cardiac defibrillator	DELA004, DELF013; DELF014; DELF016; DELF020; DELF900
Implantation of a pacemaker	DELF001, DELF007, DELF005, DELF010, DELF012, DELF015, DELF901, DELF902, DELF903, DELF904, DELF905,
Heart transplantation	DZEA00, DZEA001, DZEA002, DZEA003, DZEA004
Mechanical circulatory assistance*	EQLA008, EQLA009, EQLA010, Z994/Z998

*Include all types of mechanical mono- or biventricular assistance, with or without extracorporeal circulation.

Reference: Trombert-Paviot B, Rector A, Baud R, et al. The Development of CCAM: The New French Coding System of Clinical Procedures. Health Information Management. 2003 Mar;**31**(1):2–11.

Table S3. Inpatient prevalence of patients with cardiomyopathies per sex and per age classes.

	Gender	Age	2008	2009	2010	2011	2012	2013	2014	2015
**Dilated Cardiomyopathy**										
	All	**00-19**	270	277	306	318	304	313	332	326
	All	**20-59**	7022	7104	7243	7187	7446	7576	7614	8113
	All	**≥ 60**	18937	18636	18254	18384	19066	19971	20738	22084
	All	**All**	26229	26017	25803	25889	26816	27860	28684	30523
	Female	**00-19**	111	107	127	145	136	138	147	149
	Female	**20-59**	1829	1805	1849	1869	1963	2016	2045	2157
	Female	**≥ 60**	7254	7121	7056	7033	7330	7652	8073	8624
	Female	**All**	9194	9033	9032	9047	9429	9806	10265	10930
	Male	**00-19**	159	170	179	173	168	175	185	177
	Male	**20-59**	5193	5299	5394	5318	5483	5560	5569	5956
	Male	**≥ 60**	11683	11515	11198	11351	11736	12319	12665	13460
	Male	**All**	17035	16984	16771	16842	17387	18054	18419	19593
**Other Cardiomyopathy**										
	All	**00-19**	333	336	299	314	304	286	269	267
	All	**20-59**	3522	3310	3125	3254	3462	3544	3355	3096
	All	**≥ 60**	13422	12754	11879	11660	12504	12836	12522	12107
	All	**All**	17277	16400	15303	15228	16270	16666	16146	15470
	Female	**00-19**	138	135	108	111	127	112	101	103
	Female	**20-59**	1063	1041	985	1033	1142	1215	1181	1183
	Female	**≥ 60**	5820	5604	5203	5145	5607	5835	5921	5932
	Female	**All**	7021	6780	6296	6289	6876	7162	7203	7218
	Male	**00-19**	195	201	191	203	177	174	168	164
	Male	**20-59**	2459	2269	2140	2221	2320	2329	2174	1913
	Male	**≥ 60**	7602	7150	6676	6515	6897	7001	6601	6175
	Male	**All**	10256	9620	9007	8939	9394	9504	8943	8252
**Hypertrophic Cardiomyopathy**										
	All	**00-19**	351	312	317	338	325	313	350	404
	All	**20-59**	1865	1807	1769	1722	1682	1845	1902	2074
	All	**≥ 60**	5574	4773	4317	3988	3680	3591	3780	4089
	All	**All**	7790	6892	6403	6048	5687	5749	6032	6567
	Female	**00-19**	152	121	120	127	127	112	124	149
	Female	**20-59**	502	514	507	505	466	507	590	606
	Female	**≥ 60**	2554	2192	1912	1822	1633	1607	1688	1789
	Female	**All**	3208	2827	2539	2454	2226	2226	2402	2544
	Male	**00-19**	199	191	197	211	198	201	226	255
	Male	**20-59**	1363	1293	1262	1217	1216	1338	1312	1468
	Male	**≥ 60**	3020	2581	2405	2166	2047	1984	2092	2300
	Male	**All**	4582	4065	3864	3594	3461	3523	3630	4023
**Restrictive Cardiomyopathy**										
	All	**00-19**	109	107	117	89	74	91	80	88
	All	**20-59**	366	411	374	387	424	414	416	396
	All	**≥ 60**	893	947	943	931	1145	1311	1491	1609

	All	**All**	1368	1465	1434	1407	1643	1816	1987	2093
	Female	**00-19**	48	52	59	39	34	39	34	38
	Female	**20-59**	139	161	156	178	193	191	180	181
	Female	**≥ 60**	386	396	437	408	499	590	665	669
	Female	**All**	573	609	652	625	726	820	879	888
	Male	**00-19**	61	55	58	50	40	52	46	50
	Male	**20-59**	227	250	218	209	231	223	236	215
	Male	**≥ 60**	507	551	506	523	646	721	826	940
	Male	**All**	795	856	782	782	917	996	1108	1205
**All Type Cardiomyopathy**										
	All	**00-19**	1063	1036	1040	1060	1007	1004	1031	1085
	All	**20-59**	12833	12689	12580	12616	13089	13431	13348	13737
	All	**≥ 60**	38827	37110	35393	34963	36395	37709	38531	39889
	All	**All**	52723	50835	49013	48639	50491	52144	52910	54711
	Female	**00-19**	449	419	415	423	424	402	406	439
	Female	**20-59**	3591	3578	3566	3651	3839	3981	4057	4185
	Female	**≥ 60**	16015	15313	14608	14408	15069	15684	16347	17014
	Female	**All**	20055	19310	18589	18482	19332	20067	20810	21638
	Male	**00-19**	614	617	625	637	583	602	625	646
	Male	**20-59**	9242	9111	9014	8965	9250	9450	9291	9552
	Male	**≥ 60**	22812	21797	20785	20555	21326	22025	22184	22875
	Male	**All**	32668	31525	30424	30157	31159	32077	32100	33073

Figure S1. Flowchart of distinct patients who experienced at least one cardiomyopathy-related hospitalization from 2008 to 2015 in France.

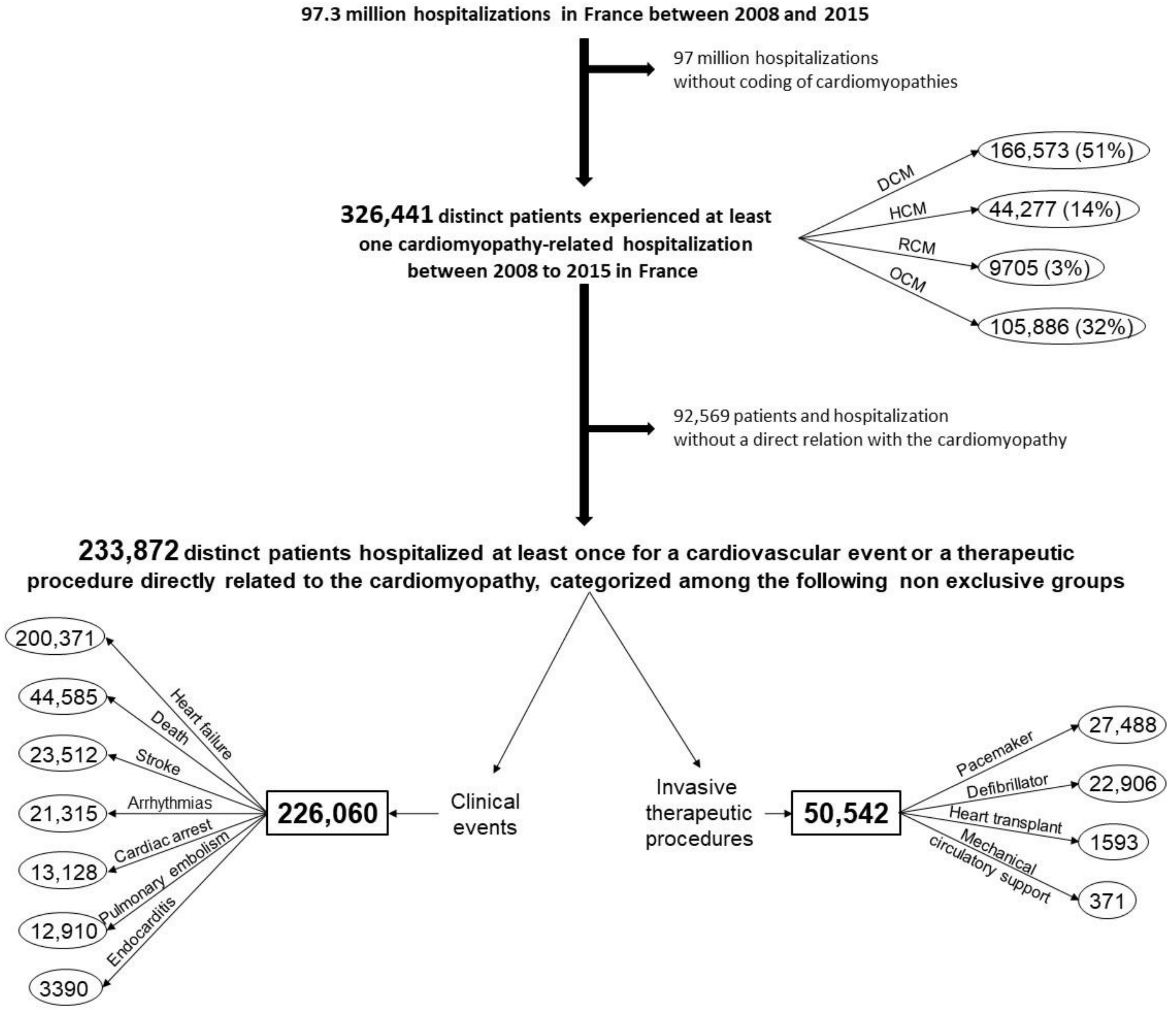

Supplement: Supplementary file 1 [file jcm-09-00920-s001.pdf]
